# Phenotype of a transient neonatal diabetes point mutation (SUR1-R1183W) in mice

**DOI:** 10.12688/wellcomeopenres.15529.1

**Published:** 2020-01-30

**Authors:** Gregor Sachse, Elizabeth Haythorne, Peter Proks, Michelle Stewart, Heather Cater, Sian Ellard, Ben Davies, Frances M. Ashcroft

**Affiliations:** 1 Department of Physiology, Anatomy and Genetics, University of Oxford, Oxford, OX1 3PT, UK; 2 Department of Physics, University of Oxford, Oxford, OX1 3PJ, UK; 3 MRC Harwell Institute, Mammalian Genetics Unit and Mary Lyon Centre, Harwell Campus, Oxfordshire, OX11 0RD, UK; 4 University of Exeter Medical School, Institute of Biomedical and Clinical Science, Barrack Road, Exeter, EX2 5DW, UK; 5 Wellcome Trust Centre for Human Genetics, University of Oxford, Roosevelt Drive, Oxford, OX3 7BN, UK

**Keywords:** KATP channel, diabetes, transient neonatal diabetes, high fat diet, mouse model

## Abstract

**Background:** The K _ATP_ channel plays a key role in glucose homeostasis by coupling metabolically generated changes in ATP to insulin secretion from pancreatic beta-cells.  Gain-of-function mutations in either the pore-forming (Kir6.2) or regulatory (SUR1) subunit of this channel are a common cause of transient neonatal diabetes mellitus (TNDM), in which diabetes presents shortly after birth but remits within the first few years of life, only to return in later life. The reasons behind this time dependence are unclear.

**Methods:** In an attempt to understand the mechanism behind diabetes remission and relapse, we generated mice expressing the common TNDM mutation SUR1-R1183W. We employed Cre/LoxP technology for both inducible and constitutive expression of SUR1-R1183W specifically in mouse beta-cells, followed by investigation of their phenotype using glucose tolerance tests and insulin secretion from isolated islets.

**Results:** We found that the R1183W mutation impaired inhibition of K _ATP_ channels by ATP when heterologously expressed in human embryonic kidney cells. However, neither induced nor constitutive expression of SUR1-R1183W in mice resulted in changes in blood glucose homeostasis, compared to littermate controls. When challenged with a high fat diet, female mice expressing SUR1-R1183W showed increased weight gain, elevated blood glucose and impaired glycaemic control, but glucose-stimulated insulin secretion from pancreatic islets appeared unchanged.

**Conclusions:** The mouse model of TNDM did not recapitulate the human phenotype. We discuss multiple potential reasons why this might be the case. Based on our findings, we recommend future TNDM mouse models employing a gain-of-function SUR1 mutation should be created using the minimally invasive CRISPR/Cas technology, which avoids many potential pitfalls associated with the Cre/LoxP system.

## Introduction

ATP-sensitive potassium (K _ATP_) channels play a pivotal role in coupling blood glucose levels to insulin secretion ( Ashcroft, 2007). A rise in glucose availability increases the intracellular ATP/ADP ratio of pancreatic beta-cells, which closes K _ATP_ channels, leading to depolarization of the beta-cell plasma membrane, calcium influx and insulin release. In the beta-cell, the K _ATP_ channels is composed of four pore-forming Kir6.2 subunits (encoded by the *KCNJ11* gene) and four regulatory SUR1 subunits ( *ABCC8*), which together form an octameric complex ( Puljung, 2018). Activating mutations in either *KCNJ11* or *ABCC8* are a common cause of neonatal diabetes (NDM) ( De Franco *et al.,* 2015; Edghill *et al.,* 2010; Flanagan *et al.,* 2009; Gloyn *et al.,* 2004). These mutations impair the ability of MgATP to close the K _ATP _channel, thereby preventing insulin secretion in response to elevation of blood glucose ( Babenko *et al.,* 2006; Gloyn *et al.,* 2004; Gloyn *et al.,* 2005; Proks *et al.,* 2006).

Neonatal diabetes is characterised by a low birth weight and diabetes that presents within the first six months of life. It has an incidence of around 1 in 100,000 live births ( Iafusco *et al.,* 2012; Slingerland *et al.,* 2009). In many cases the diabetes is permanent (PNDM). However, in some patients the disease exhibits a remitting relapsing phenotype in which diabetes presents shortly after birth but remits within the first few years of life only to return again in later life (transient neonatal diabetes, TNDM) ( Flanagan *et al.,* 2007). Why diabetes remits, only to later relapse, remains unclear. It has been proposed that it results from a reduced insulin requirement at the time of remission, or some mechanism at the level of the beta-cell or the whole organism that compensates for the genetic defect ( Flanagan *et al.,* 2007).

Neonatal diabetes is caused by a number of different genes and TNDM has been linked to both the K _ATP_ channel and to an abnormality of chromosome 6q24 ( Murphy *et al.,* 2008). A mouse model of the latter suggests this has a strong developmental component ( Ma *et al.,* 2004). However, the molecular mechanisms underlying remision and relapse of TNDM due to K _ATP_ channel mutations is unknown. In an attempt to elucidate these, we generated a mouse that expresses a common K _ATP_ channel mutation causing TNDM (SUR1-R1183W). Here we report data on both the generation of the model and analysis of its phenotype.

## Results

**Figure 1.**
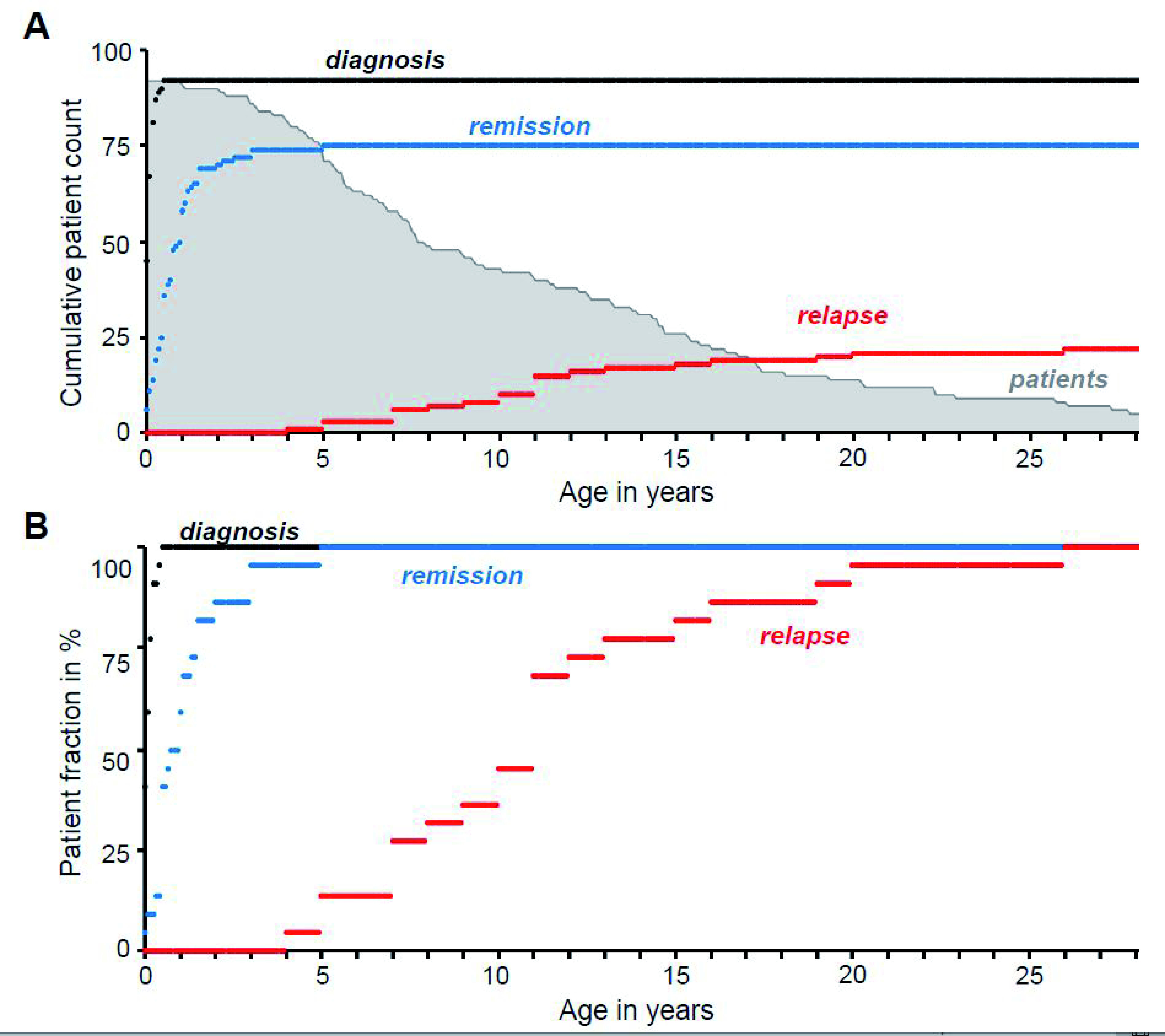
Time course of K _ATP_ channel associated transient neonatal diabetes (TNDM). **A**. Diagnosis, remission and relapse of TNDM plotted over time as the cumulative number of patients up to a given age (n = 92): i.e. the number of remission/relapse cases at any given age also includes patients younger than that age who have remitted or relapsed. The number of patients in our cohort of a given age is shown in light gray. **B**. Diagnosis, remission and relapse of TNDM plotted over time as a percentage of all patients with known relapse (n = 22). Diabetes onset is typically within the first few months of life (black trace, median age = four weeks), followed by rapid remission (blue trace) at a median age of 32 weeks. The rate of relapse is much slower and more gradual (red trace), with a median age of 11 years. Only patients with mutations in K _ATP_ channel genes *KCNJ11* (Kir6.2) and *ABCC8* (SUR1) known to cause TNDM are shown.

Figure 1A shows the time of diabetes diagnosis, remission and relapse in the Exeter cohort of 92 TNDM patients with activating K _ATP_ channel mutations. Classification as TNDM is based on either i) diagnosis of diabetes before the age of six months that has remitted or ii) the presence of a known TNDM mutation in a newly diagnosed patient. For the 22 patients that had reached relapse stage at the time of study, the median age of diagnosis was four weeks, that of remission 32 weeks and the median age of relapse was 11 years ( Figure 1B). To explore why some activating K _ATP_ channel mutations cause transient, rather than permanent, diabetes, we generated a mouse expressing an inducible TNDM SUR1 mutation (SUR1-R1183W) specifically in pancreatic beta cells. This mutation was chosen as it was the most common mutation associated with TNDM in this cohort. This mutation usually results in TNDM ( Flanagan *et al.,* 2007) but PNDM has rarely been described ( Hashimoto *et al.,* 2017). In our cohort, 11 patients carry the R1183W mutation, with an additional two carrying a mutation at the same residue (R1183Q). The majority of these patients showed the typical feature of remitting and relapsing TNDM. All thirteen R1183W/Q patients are over the age of three. For five of these patients, we have no reports of remission, which either reflects PNDM, a period of remission that went unnoticed, or a lack of follow-up data. Of the other eight patients who had gone into remission, we know of at least five who subsequently relapsed, with two years being the earliest age and 20 years the latest age of the reported relapse.

### The SUR1-R1183W mutation impairs ATP inhibition

**Figure 2.**
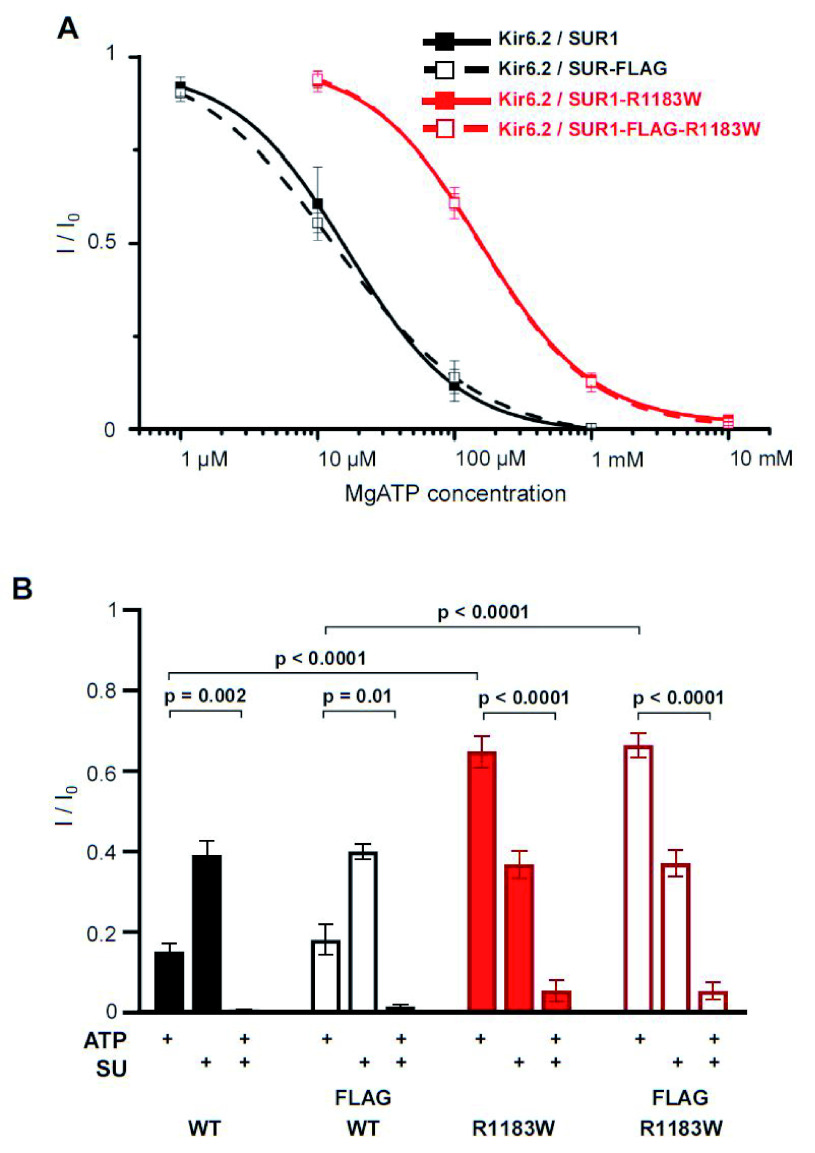
The SUR1-R1183W mutation impairs K _ATP_ channel inhibition by intracellular MgATP. **A**. Concentration-response relation for MgATP inhibition of Kir6.2/SUR1 (wild-type), Kir6.2/SUR1-R1183W, Kir6.2/SUR1-FLAG and Kir6.2/SUR1-R1183W-FLAG K _ATP_ channels. Current (I) is expressed as a fraction of that in the absence of MgATP (I _0_). n=5 patches per group (from at least two independent transfections). Insertion of an extracellular FLAG tag (open symbols) did not affect MgATP sensitivity. SUR1-R1183W, wild-type SUR1 and their respective 3-FLAG-tagged versions were transiently expressed with Kir6.2 in HEK293 cells and K _ATP_ currents measured in excised inside-out membrane patches. **B**. Same experiment as in A but comparing the mean fractional current I/I _0 _in the presence of 100 µM MgATP (ATP), 30 µM gliclazide (SU) or both. n=5 per group. Error bars denote SEM. Welch’s t-test was used to test for statistical significance.

We first characterised the ATP sensitivity of SUR1-R1183W mutant channels in excised inside-out patches by heterologous expression of mouse SUR1-R1183W with wild-type Kir6.2 in HEK293 cells. Figure 2A shows that the SUR1-R1183W mutation causes a significant reduction in the ability of MgATP to inhibit K _ATP_ channel currents, half maximal inhibition (IC _50_) being shifted from 21±6µM (n = 5, wild-type) to 158±13µM (n=5, homomeric Kir6.2/SUR1-R1183W). Introduction of a 3-FLAG tag between residues V1049 and L1050 of mouse SUR1 did not change the MgATP sensitivity of either wild-type (IC _50, _15±3 µM, n=5) or mutant channels (IC _50, _159±21 µM, n=5). The ability of the sulphonylurea gliclazide to block the K _ATP_ current was not significantly affected by the SUR1-R1183W mutation ( Figure 2B), which helps explain why patients with this mutation can successfully transfer from insulin to sulphonylurea therapy ( Rafiq *et al.,* 2008). There was also no effect of the 3-FLAG tag on MgATP sensitivity ( Figure 2B).

### SUR1-R1183W mouse model generation

**Figure 3.**
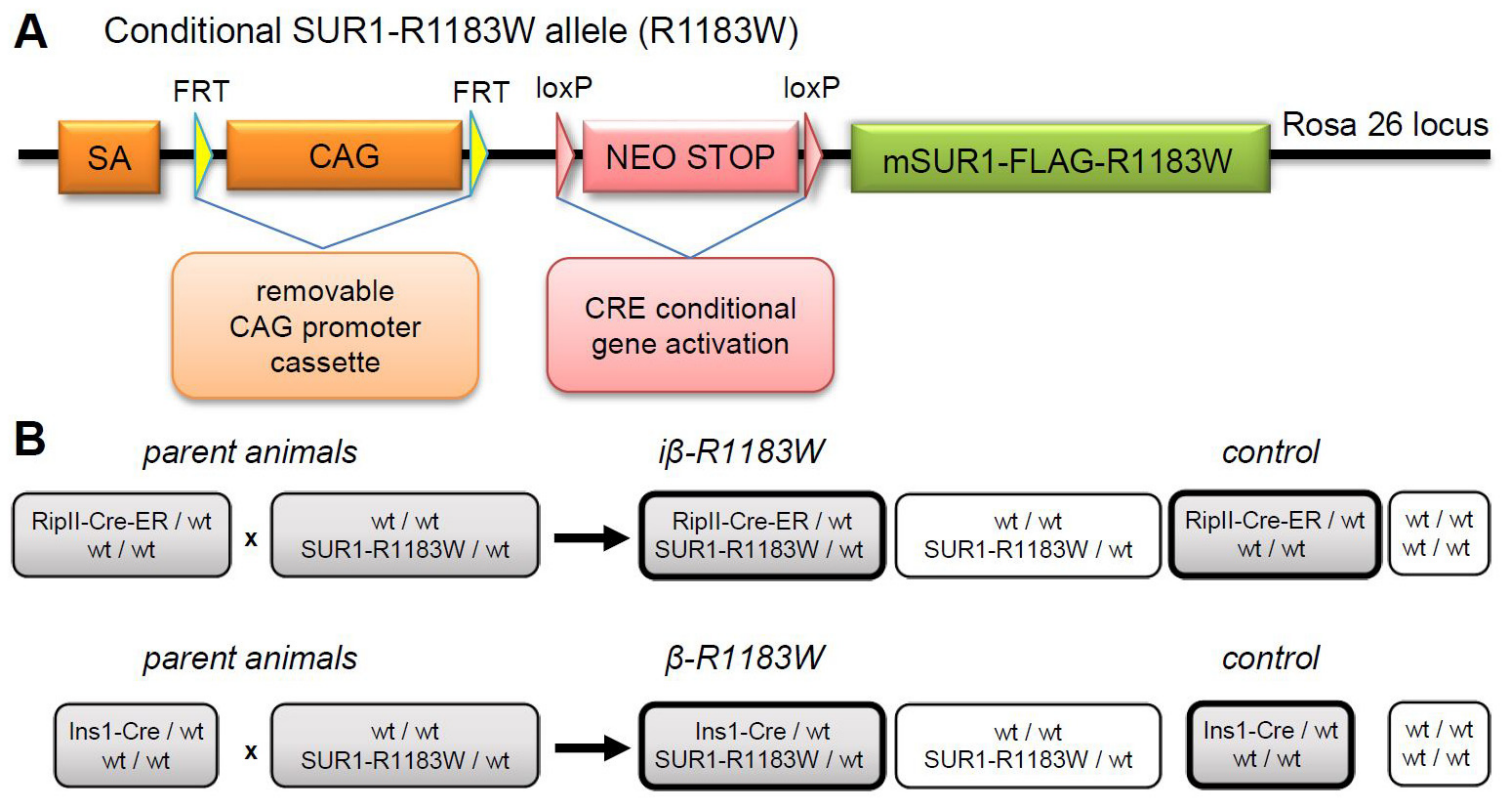
Genetic construct and breeding strategy. **A**. cDNA for mouse SUR1 containing the R1183W mutation and an extracellular 3-FLAG tag sequence was integrated into the Rosa26 locus using a shuttle system optimized for overexpression studies ( Dolatshad *et al.,* 2015). No expression of SUR1-R1183W occurs until the upstream STOP codon is deleted by Cre recombinase. Expression is then driven by the strong CAG promoter or alternatively, after excision of the CAG module with Flp recombinase, by the medium-strength endogenous Rosa26 promoter. The CAG promoter was used for all studies reported here. **B**. Breeding scheme for experimental animals (for explanation see main text).

We next generated mice expressing a transgene cassette consisting of mouse SUR1 containing both the R1183W mutation and the 3-FLAG tag. The tag was used to distinguish between mutant and wild-type channel protein. This construct was inserted downstream of a floxed stop cassette and an FRT-removable CAG promoter, and the transgene cassette was then inserted into the Rosa26 locus receiver site ( Figure 3A) using a gene shuttle system optimized for overexpression studies ( Dolatshad *et al.,* 2015). 

Mice carrying the floxed-stop SUR1-R1183W allele were crossed with one of two Cre strains to drive beta-cell specific expression ( Figure 3B). First, for tamoxifen-inducible beta-cell expression, mice were crossed with RipII-Cre-ER mice ( Herrera, 2000), generating heterozygous RipII-Cre-ER/SUR1-R1183W mice. We refer to these as iβ-R1183W mice. Second, for constitutive beta-cell expression from before birth, mice were crossed with Ins1-Cre mice ( Thorens *et al.,* 2015), generating heterozygous Ins1-Cre/SUR1-R1183W (β-R1183W) mice.

**Figure 4.**
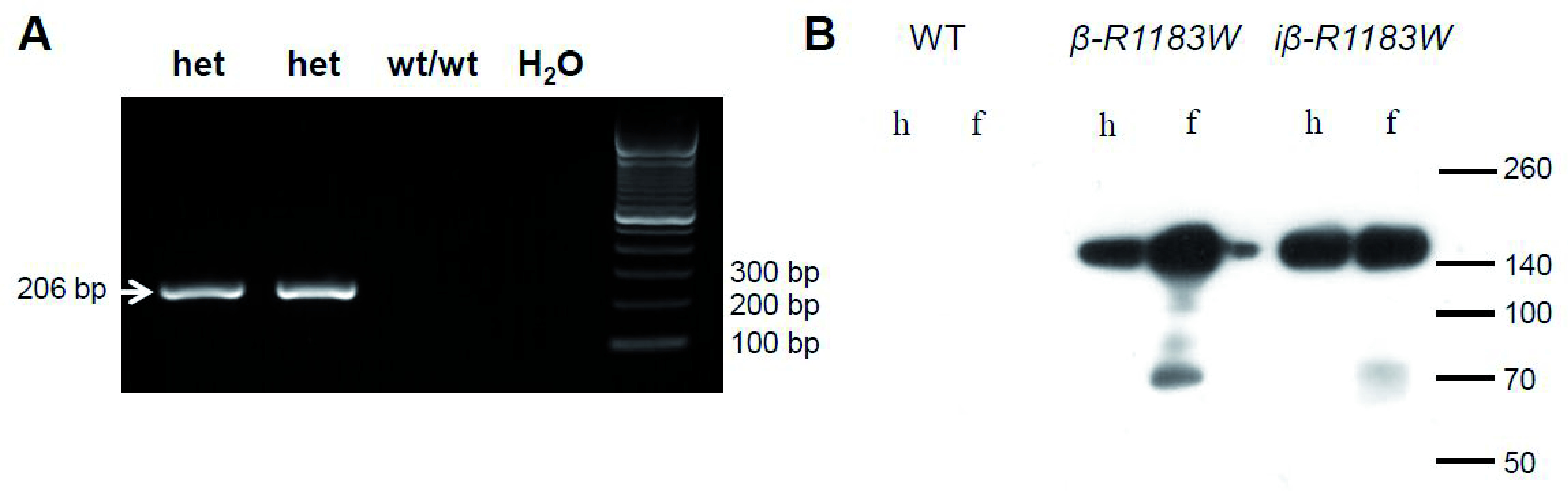
R1183W allele validation. **A**. Proof of germ line transmission by conventional PCR. An R1183W allele specific 206 bp band was detected in R1183W/wt F1 animals (het) but not in littermate controls (wt/wt). DNA ladder shown on the right. Germ line transmission and integration site were additionally confirmed using copy number quantitative real time PCR (details see Methods). **B**. SUR1-FLAG-R1183W protein expression in pancreatic islets, confirmed using anti-FLAG western blotting. Islets were isolated from wild-type controls, β-R1183W mice, and iβ-R1183W mice either by handpicking (h) or by Ficoll cushion centrifugation enrichment (f). Three animals were tested per genotype. Molecular weights (in kDa) are indicated on the right.

Successful generation of the SUR1-R1183W allele was confirmed using conventional PCR ( Figure 4A). Germline transmission and correct integration were confirmed by copy number quantitative real-time PCR (see Methods). Protein expression in pancreatic islets was confirmed by anti-FLAG western blotting, for both iβ-R1183W mice after tamoxifen induction and for β-R1183W animals ( Figure 4B).

### Phenotype of iβ-R1183W mice following induction at seven weeks

**Figure 5.**
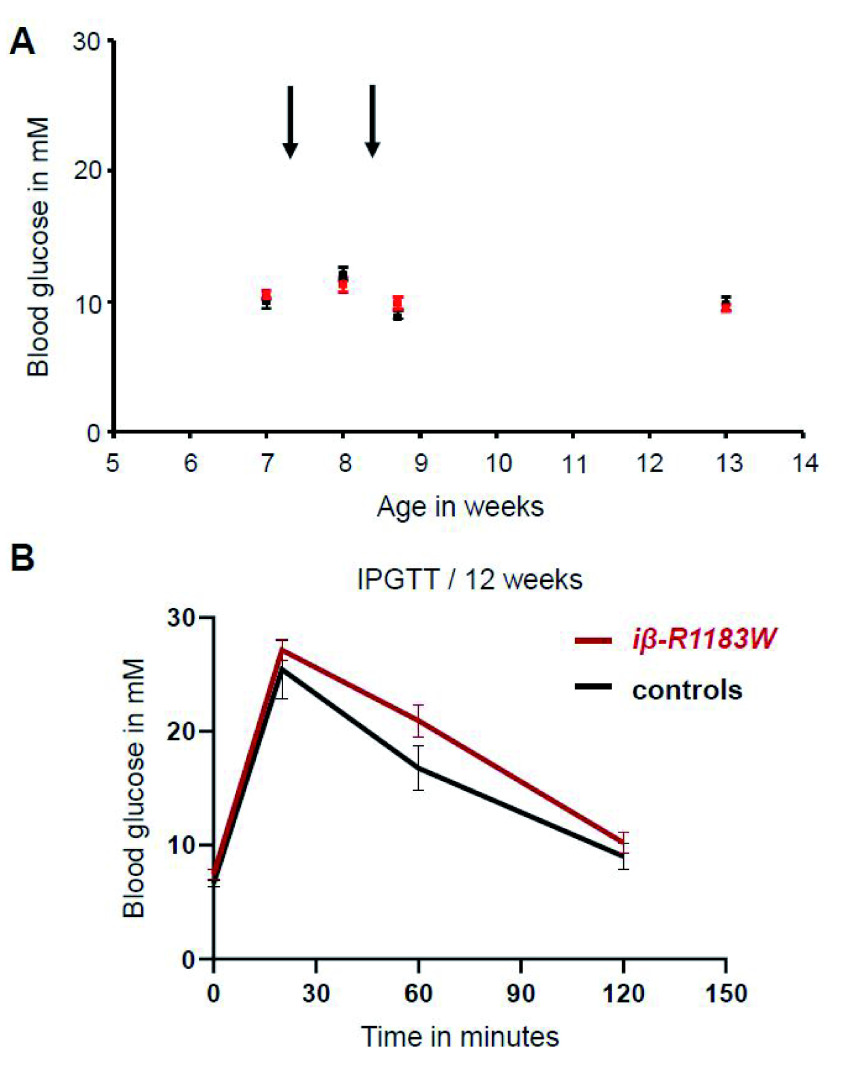
Fed blood glucose and glucose tolerance in iβ-R1183W mice. **A**. Fed blood glucose levels for β-R1183W mice (red, n=11) and littermate controls (black, n=4) measured in the afternoon (light phase) at the indicated age. Tamoxifen was injected twice (arrows) to induce transgene expression. **B**. Intraperitoneal glucose tolerance test for control (black, n=4) and iβ-R1183W (R1183W, red, n=8) mice, measured at 12 weeks of age. Differences were not statistically significant (Welch’s t-test). Error bars denote SEM. Due to the small cohort size, data from male and female mice were combined (female:male ratio of 1:1 in all groups).

Tamoxifen injection of seven-week-old experiment-naïve iβ-R1183W mice did not affect fed blood glucose levels ( Figure 5A), despite protein expression in islets ( Figure 4). In an attempt to induce SUR1-R1183W expression in as high a percentage of beta-cells as possible, a second maximal-dose tamoxifen injection was given at eight weeks of age. However, no change in fed blood glucose levels was detected, either immediately after the second tamoxifen injection or five weeks later at 13 weeks of age ( Figure 5A). Baseline body weight at first injection was approximately 22–25g (females) & 28–31g (males); no adverse effects were noted during, or after, either injection. For housing conditions, feeding and strain background: see methods section.

At 12 weeks of age, the cohort underwent an intra-peritoneal glucose tolerance test (IPGTT) after an overnight fast ( Figure 5B). iβ-R1183W mice showed slightly elevated blood glucose both after fasting and following a glucose challenge. Fasting blood glucose was 7.45±0.43mM (n=8) for iβ-R1183W mice and 6.63±0.31mM (n=4) for Cre-only control mice, and the area under the curve (AUC) was 2244±217mM*min and 1939±217mM*min, respectively (mean±SEM). However, these differences were not statistically significant (p=0.15 and p=0.20, respectively). There was also no difference in body weight between genotypes at 12 weeks of age: 27.8±1.5g (n = 8) for iβ-R1183W and 27.6±2.6g (n=4) for controls. No adverse effects were noted during, or after, the IPGTT procedure.

### Phenotype of iβ-R1183W mice with constitutively active SUR1-R1183W

In TNDM patients, diabetes arises early after birth and remits within the first few years of life ( Figure 1). To screen for potential blood glucose changes in newly weaned animals, we used a constitutively active Ins1-Cre allele, which drives expression in all insulin-expressing cells early in life and has a ~98% recombination efficiency ( Thorens *et al.,* 2015). However, we found no difference in the fed blood glucose level in young animals (four weeks of age), which was 11.9±0.4mM (n=8) in β-R1183W mice and 12.7±0.9mM (n=8) in Ins-1-Cre controls (p=0.39, Welch’s t-test). Body weight, first measurement directly after weaning, was approximately 15–17g (females) & 18–20g (males); no adverse effects were noted during, or after, blood glucose measurements. For housing conditions, feeding and strain background: see methods section.

### Increased weight gain in female β-R1183W *mice on a high fat diet (HFD)*

**Figure 6.**
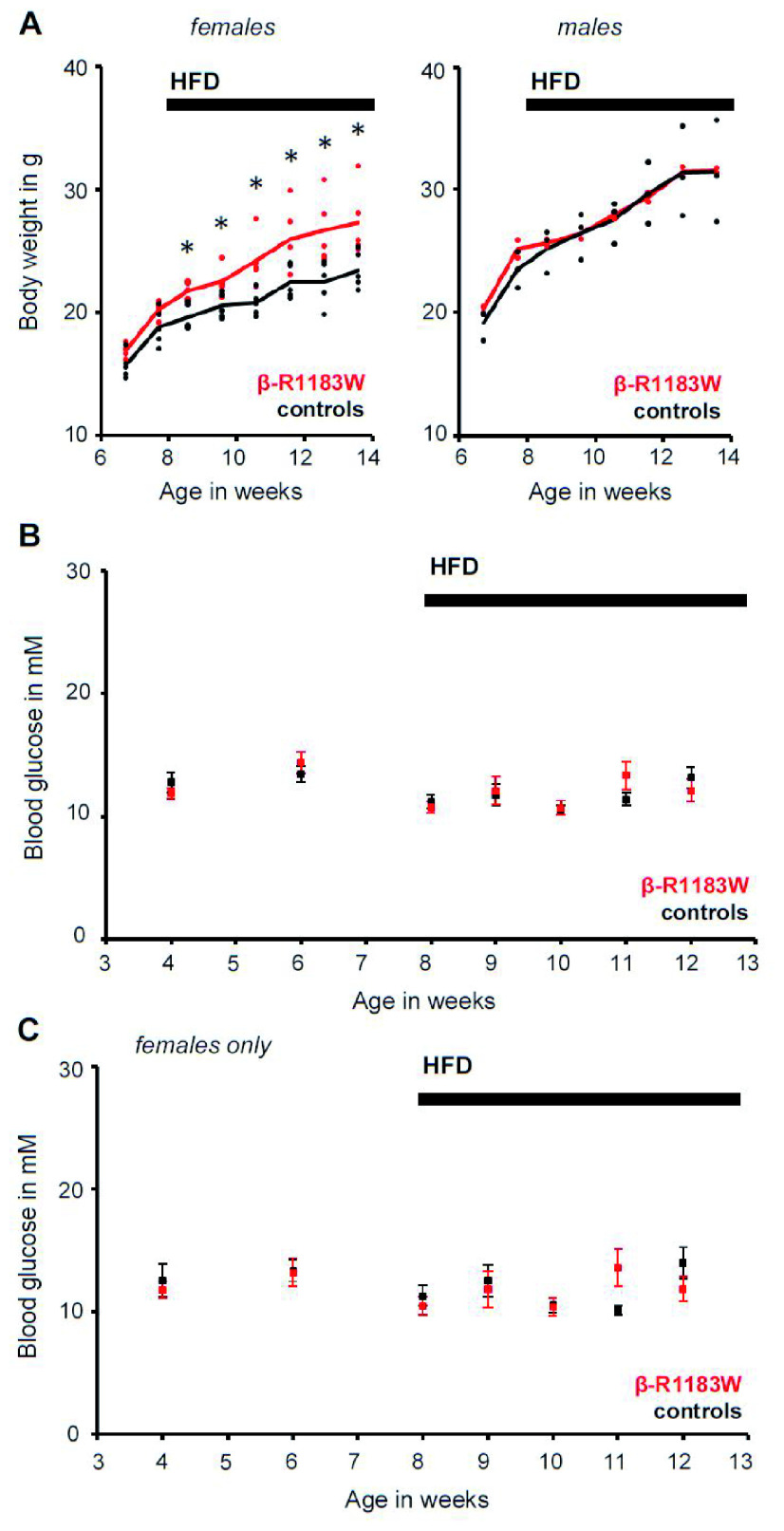
Body weight and fed blood glucose β-R1183W mice. **A**. Body weight of control (black, n=5) and β-R1183W (R1183W, red, n=10) mice on a standard and high fat diet (HFD). The HFD was started at eight weeks of age. Data shown separately for both genders. The data points represent individual animals and the lines connect the corresponding genotype average. *: p < 0.05 (Welch’s t-test). **B**, **C**. Time dependence of fed blood glucose measured in the afternoon (light phase), for both male and female mice ( **B**) and for female mice only ( **C**). A HFD was introduced at week eight. The data points represent individual animals and the error bars denote SEM. Error bars are smaller than symbols for some data. There was no statistically significant difference between control and β-R1183W mice (Welch’s t-test).

We explored the possibility that an additional metabolic challenge was required to uncover the phenotypic effects of the SUR1-R1183W gain-of-function mutation. β-R1183W mice and littermate controls were given a HFD (for composition see Methods), starting at eight weeks of age. Gender-specific analysis revealed that female β-R1183W animals showed consistently higher weight gain than controls after the start of the HFD ( Figure 6A). However, the relative increase in weight did not correlate with any changes in fed blood glucose levels, either in the whole cohort ( Figure 6B) or when considering female animals alone ( Figure 6C).

### Reduced glucose tolerance and loss of insulin sensitivity in female β-R1183W mice on a HFD

**Figure 7.**
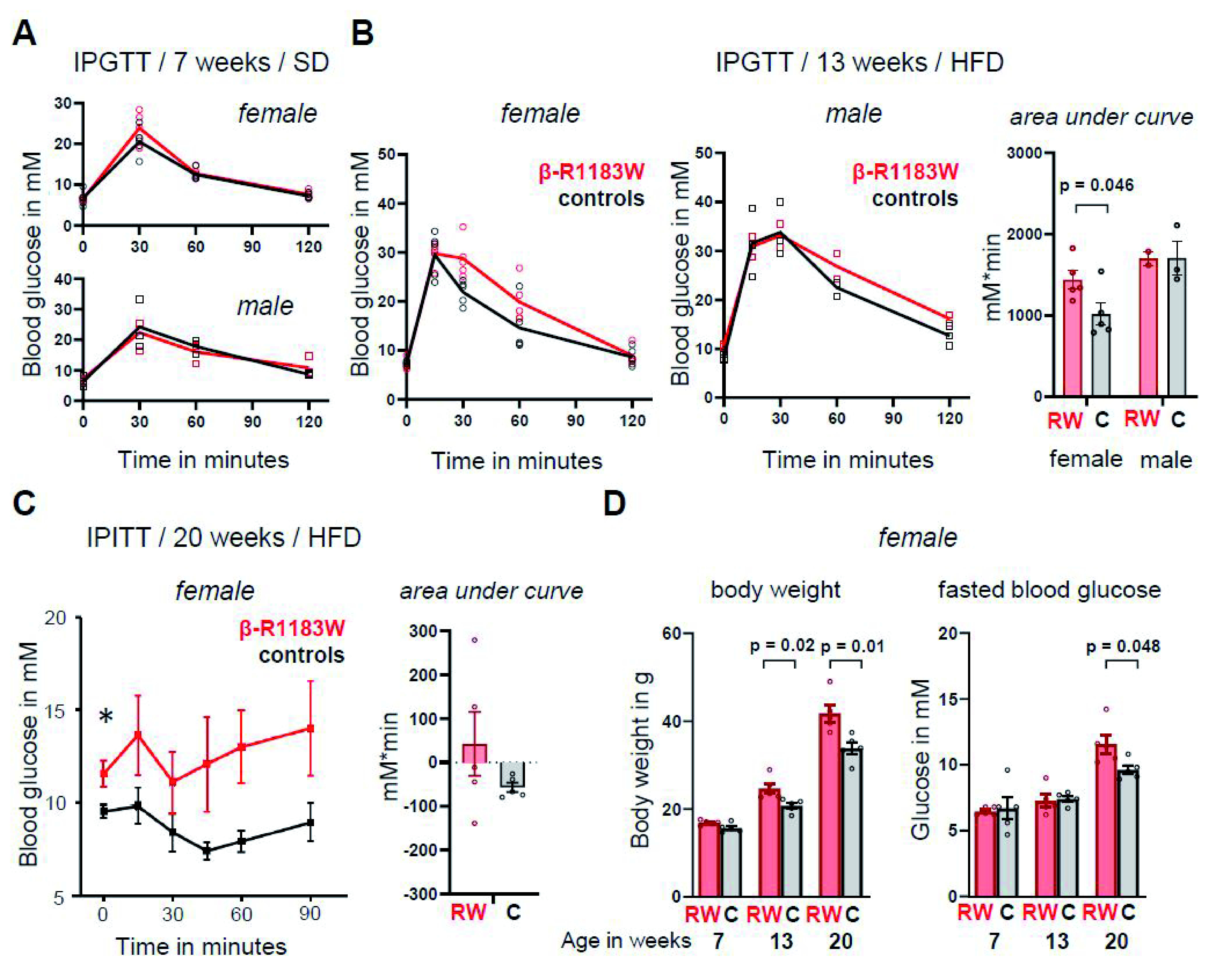
Blood glucose control in β-R1183W on a standard and high fat diet. **A**. Intraperitoneal glucose tolerance test (IPGTT) in seven-week-old female (above) and male (below) control (black) and β-R1183W (red) mice fed a standard diet (SD). The data points indicate individual animals. The lines connect the mean data at each timepoint. **B**. IPGTT in 13-week-old female (left) and male (middle) control (black) and β-R1183W (red) mice. Animals were fed a standard diet (SD) for eight weeks followed by five weeks on a high fat diet (HFD). Right, the area under curve (AUC) data for control (C) and β-R1183W (RW) female and male mice. AUC calculated as the area above the fasting blood glucose baseline. The data points indicate individual animals. Error bars denote SEM. **C**. Intraperitoneal insulin tolerance test (IPITT, left) and corresponding AUC (right), measured for 20-week-old control and β-R1183W female and male mice, after 12 weeks on HFD. **D**. Time dependence of body weight (left) and fasting blood glucose (right) for β-R1183W mice (RW, n=5) and Cre-only littermate controls (C, n=5). p values calculated with Welch’s t-test. The data points indicate individual animals. Error bars denote SEM.

Like iβ-R1183W mice, β-R1183W mice had normal glucose tolerance at seven weeks of age when fed a standard diet ( Figure 7A). However, after five weeks of a HFD (from 8 to 13 weeks of age), female iβ-R1183W mice showed reduced glucose tolerance in an IPGTT ( Figure 7B). No adverse effects were noted during, or after, IPGTTs. Several weeks after the IPGTTs, the small number of available male animals was reduced as one animal had to be culled because of fight wounds (incident unrelated to experimental procedures). We therefore focused on female mice in the following experiments.

After 12 weeks on a HFD, at 20 weeks of age, weight differences between the genotypes became substantial (β-R1183W 83W = 41.8±2.0g, Ins1-Cre = 33.9±1.4g; p=0.01; n=5 each group). This was accompanied by an increase in fasting blood glucose ( Figure 7C and 7D). Importantly, changes in body weight preceded the changes in fasting blood glucose levels in female β-R1183W mice ( Figure 7D). During an intra-peritoneal insulin tolerance test (IPITT) at 20 weeks of age, Ins1-Cre control mice on a HFD exhibited a small and consistent reduction of blood glucose concentration, but β-R1183W mice showed highly variable responses (F-test for variances: p = 0.002, Figure 7C).

**Figure 8.**
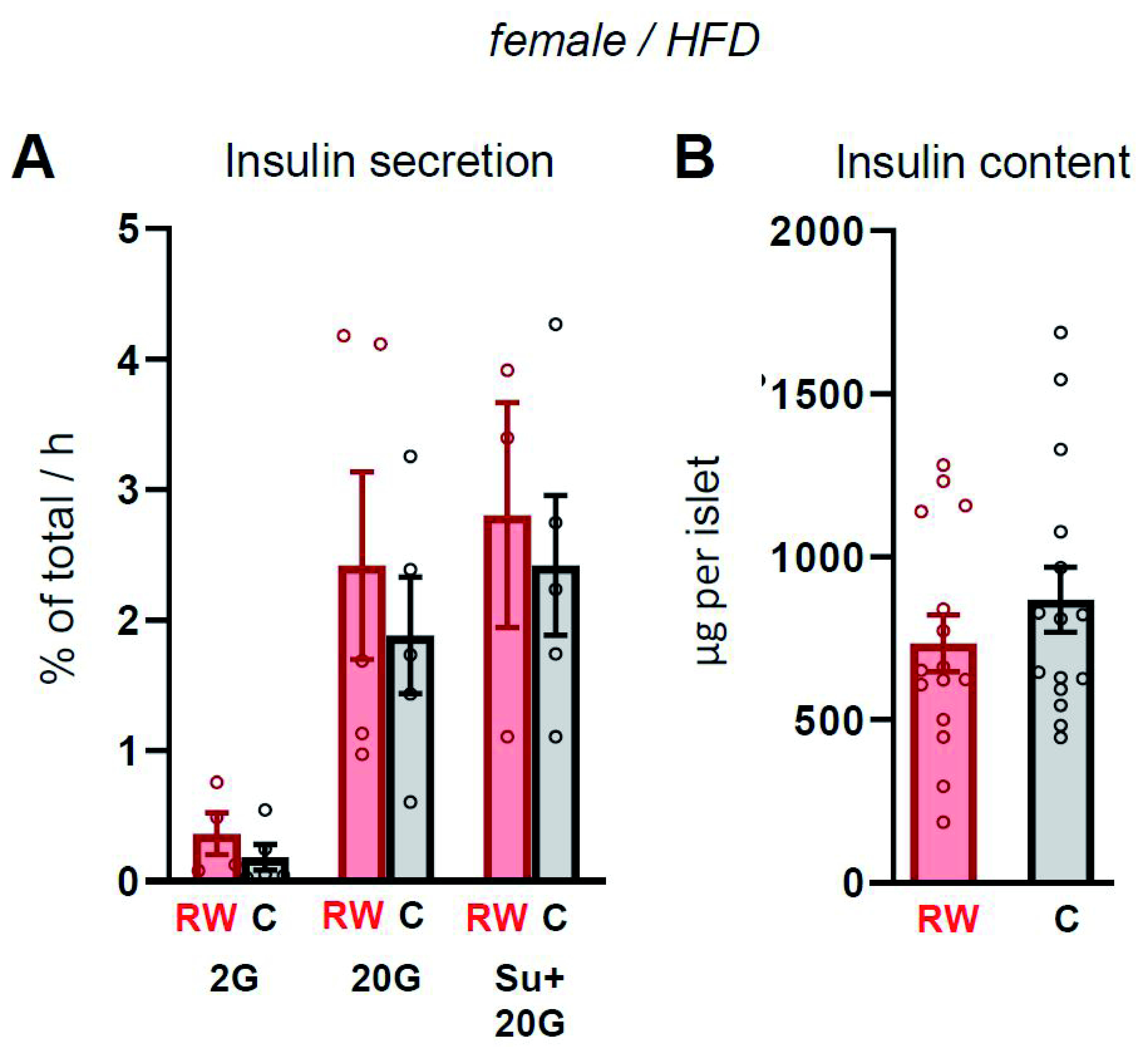
Insulin secretion is unchanged in β-R1183W islets. **A**. Insulin secretion from Cre-only control (C) and β-R1183W (RW) islets measured by batch incubation for 1 hour at 2 mM glucose (2G), 20 mM glucose (20G) or in the presence of 20 mM glucose and 1 µM glibenclamide (SU), n=5 mice per genotype. Secretion is expressed as a percentage of insulin content. All data come from 20-week-old female mice on a HFD (those shown in Figure 6 and Figure 7). Error bars are mean ± SEM. **B**. Insulin content of islets shown in A. Each data point indicates a different batch of islets (n=15 per group). Differences were statistically not significantly (Welch’s t-test). Error bars denote SEM.

We isolated pancreatic islets from β-R1183W mice and Ins1-Cre control littermates (on a HFD) and tested glucose-stimulated insulin secretion in batch incubations. However, neither insulin secretion ( Figure 8A) nor total insulin content ( Figure 8B) differed significantly between genotypes.

## Discussion

Mice were successfully genetically modified to conditionally express a common mutation causing transient neonatal diabetes mellitus - SUR1-R1183W. Expression was controlled using the Cre/LoxP system, enabling both inducible and constitutive expression ( Figure 3). Mice expressing SUR1-R1183W either inducibly or constitutively had unaltered blood glucose levels and normal glycaemic control, both post-weaning and in adult life when fed a standard diet ( Figure 5, Figure 6 and Figure 7). Thus, the animal model does not appear to recapitulate the patient phenotype.

After five weeks on a HFD, glycaemic control of female β-R1183W mice was more severely impaired than that of their control littermates ( Figure 7B). After 12 weeks on a HFD, their fasting blood glucose levels were significantly above those of littermate controls ( Figure 7C). However, no statistically significant changes in insulin secretion in response to 2mM or 20mM glucose were detected ( Figure 8). Female β-R1183W mice also showed a greater weight gain in response to the HFD than the Cre-only controls ( Figure 6A). Notably, changes in weight gain preceded the change in fasting blood glucose ( Figure 7D). Their response to an insulin tolerance test was highly variable but appeared to be reduced ( Figure 7C). Thus, changes in insulin resistance might underlie the impaired glycaemic control.

There are multiple potential causes for our failure to observe impaired insulin secretion and diabetes in β-R1183W and iβ-R1183W mice. Some of these would prevent presentation of a diabetic phenotype and others would mask a diabetic phenotype (especially a mild one).

First, the ATP sensitivity of SUR1-R1183W mutant channels may not have been impaired as strongly in mouse beta-cells as in the patients’ beta-cells, or as in heterologous cells ( Figure 2). We measured the ATP sensitivity of homomeric channels in HEK293 cells, whereas all patients are heterozygous and the mice are hemizygous (i.e. they carry two copies of the wild-type gene and one copy of the mutant gene). Thus, the channel ATP-sensitivity will certainly be less in both mouse and human beta-cells. It is possible that wild-type and mutant channel subunits express in a different ratio in patients and our mice. While it is known that a single mutant Kir6.2 subunit is sufficient to affect the ATP sensitivity of the whole K _ATP_ channel, each SUR1 subunit seems to independently and incrementally contribute to overall channel regulation ( Puljung *et al.,* 2019). It is therefore possible that the ratio of mutant to wild-type subunits in our mice is too small to alter the channel ATP sensitivity sufficiently.

Although substantial expression of SUR1-R1183W at the protein level was detected by western blot using an antibody against the 3-FLAG tag ( Figure 4B), we were unable to compare the relative levels of wild-type and mutant protein because we could not probe for wild-type SUR1 this way. Thus, in retrospect, it would have been beneficial to have measured the ATP sensitivity of the K _ATP_ channel in both β-R1183W and iβ-R1183W mouse beta-cells to determine how much it was altered. This would help elucidate why no overt changes in glucose-induced insulin secretion were observed ( Figure 8). 

A second possibility is that transient diabetes might have occurred directly after birth in INS1-Cre SUR1-R1183W, and mice measured directly post weaning might already have been in remission. Although this cannot apply in the case of iβ-R1183W mice, as gene expression was induced in adult life, these animals may have missed the age window of phenotypical susceptibility by the time of induction. 

Third, it could be argued that Cre-controlled overexpression of SUR1-R1183W might be sub-optimal due to mosaic expression or potential compensation effects. This seems unlikely, however, for several reasons. First, beta-cell specific overexpression using RipII-Cre-ER has previously been employed successfully to create a permanent NDM mouse model ( Brereton *et al.,* 2014; Girard *et al.,* 2009). Second, electrical coupling between beta-cells is expected to compensate for mosaicism ( Nguyen *et al.,* 2014; Rocheleau *et al.,* 2006; Zhang *et al.,* 2008). Further, the Ins1-Cre allele has a ~98% recombination efficiency ( Thorens *et al.,* 2015).

A fourth possibility stems from the fact that Kir6.2/SUR1 K _ATP_ channels are not only expressed in the pancreatic beta-cell. They are also found in many brain neurones, including glucose-sensing neurones and those that control appetite, food intake, and motor function ( Clark *et al.,* 2010; Karschin *et al.,* 1997; Mourre *et al.,* 1990; Sohn, 2013). It is therefore possible that neuronal expression of SUR1-R1183W contributes to the human phenotype. This would have been missed in both our mouse models as the mutant channels were only expressed in beta-cells. We also cannot exclude the possibility that expression of mutant channels in the L and K cells of the gut ( Reimann *et al.,* 2008), influences the phenotype (for example, via altered secretion of gut hormones).

There are also several factors that may mask or obscure mild phenotypic differences.

First, genetic background differences between mice and human patients might explain the discrepancy in phenotype. Mice have been successfully used to model permanent neonatal diabetes in the past ( Brereton *et al.,* 2014; Girard *et al.,* 2009; Kautz *et al.,* 2012; Koster *et al.,* 2000; Remedi *et al.,* 2009; Watanabe *et al.,* 2009). However, it is unclear if the mouse is a good model for transient NDM. In general, transient NDM mutations result in a weaker K _ATP_ channel gain-of-function than permanent NDM mutations, and not all carriers of the mutations develop transient NDM. Consequently, a transient NDM mouse model might be more successful on a more permissive genetic background like the C57BL/6J strain. Due to the Cre/LoxP system used, all experimental mice in this study were on a C57BL/6J-C57BL/6N F1 hybrid background, and therefore might be less susceptible to diabetes, due to hybrid vigor effects ( Khan, 2013).

Second, statistical variance between individual animals is always a concern when phenotypic differences between animals are small. Although our experimental protocols were optimized to minimize variability, it would be possible to improve the experimental approach further by synchronizing cohort breeding, combined with randomized re-shuffling of litters. An added advantage of this is litter size equalization, further reducing environmental differences between animals.

Finally, traditional techniques for generation of knock-in and transgenic mice bring with them the risk of potential confounding effects, mediated by gene variants that are close to the genetically modified locus on the same chromosome ( Sigmund, 2000). Differences in local genetic background that originate from the mouse genetic modification process are very difficult to get rid of, and will be unequally distributed between experimental groups because they are genetically linked to the modified gene locus that defines the experimental group (in this case the SUR1-R1183W mini gene in the Rosa26 gene locus). This may result in significantly confounding genotype-phenotype differences between experimental groups that are unrelated to the intended genetic modification. It is possible that this is responsible for increased weight gain seen for Ins1.SUR1-R1183W female mice on a HFD ( Figure 6A). Body weight phenotypes are extremely common, with about one in three knockout mouse strains exhibiting altered body weight ( Reed *et al.,* 2008).

An alternative approach that would avoid many of the pitfalls outlined above is to produce a targeted point mutation at the endogenous genomic SUR1 locus using CRISPR/Cas9 technology ( Inui *et al.,* 2014). This is a genetically minimal invasive approach that avoids introduction of any genetic confounding factors. Moreover, because the mutant subunit is expressed from the endogenous promoter (as in the patients), this ensures non-mosaic expression, an endogenous expression pattern (in time and space), endogenous regulation of mutant gene expression in response to physiological stimuli, and the natural stoichiometric ratio of mutant to wild-type SUR1 protein.

## Methods

### Animal experimental groups

The generation of iβ-R1183W and β-R1183W animals is described in the Results section. Animals were assigned to experimental groups based on their genotype, Cre-only littermates served as control for both iβ-R1183W and β-R1183W animals ( Figure 3B). Power calculations for animal experiments (blood glucose control, glucose stimulated insulin secretion) were based on previous data from mice with beta-cell specific expression of a similar K _ATP_-channel gain of function mutation ( Girard *et al.,* 2009). Based on effect sizes and standard deviations published by Girard *et al.*, small group sizes of N = 3 were calculated to provide sufficient statistical power (1-β > 90%) at a false type I error rate α < 5%. Surplus animals from cohort breeding beyond the core N = 3 were included in the experimental groups as contingency in case of unexpected animal deaths or smaller than expected effect sizes. Facilities and protocols allowed for processing of such additional animals without a significant increase in cost or effort. Order of treatment or measurement was random and experimenters were blinded to animal genotype.

### Ethical statement

All animal studies were licensed by the Home Office under the Animals (Scientific Procedures) Act 1986 Amendment Regulations 2012 (SI 4 2012/3039), UK (licence number PPL 30/3198). All studies were approved by the local Animal Welfare and Ethical Review Body at MRC Harwell, under the ethical guidelines issued by the Medical Research Council (Responsibility in the Use of Animals for Medical Research, July 1993) as part of the licensing process. All mice were maintained in accordance with the UK Home Office Welfare guidelines and project licence restrictions. Mice were euthanized by Home Office Schedule 1 methods.

Exeter patient cohort data were collected in accordance with the Declaration of Helsinki, as revised in 2000. Informed consent was obtained from all patients, with parental consent given on behalf of children. No additional ethical approval was required for secondary analysis of the data as patient data was queried in de-identified form. For any questions related to the original data collection please contact the Diabetes Genes genetics laboratory at the Royal Devon & Exeter NHS Foundation Trust ( https://www.diabetesgenes.org/).

### Exeter patient cohort data source and analysis

Exeter patient cohort query data was received in non-identifiable form from the Genetic Beta Cell Research Bank of the Diabetes Genes genetics laboratory (Exeter, UK). Queried fields were: “Kir62Result”, “ABCC8Result”, “DOB”, “Age Diagnosis (weeks)”, “Age Remission (weeks)”, “Age Relapse (years)”. Data are available in full on Figshare (see *Data availability* section) and was used for the generation of Figure 1 using Microsoft Excel 2010. DOB (Date of birth) data were converted into “Age at query (years)” as part of the de-identification process. For access to the Genetic Beta Cell Research Bank database please contact: Prof Sian Ellard ( sian.ellard@nhs.org), Department of Molecular Genetics, RILD Level 3, Royal Devon and Exeter NHS Foundation Trust, Barrack Road, Exeter, EX2 5DW, UK.

### Animal husbandry

Animals were housed under specific opportunistic pathogen-free (SOPF) conditions, in individually ventilated cages. Mice were kept under controlled light (light 7am–7pm, dark 7pm–7am), temperature (21±2 °C) and humidity (55±10%) conditions. They had free access to water (9–13 ppm chlorine), and were fed ad libitum on a standard diet (Rat and Mouse No. 3 Breeding diet, RM3; Diatex Int. Ltd., Witham, UK) containing 11.5 kcal% fat, 23.93 kcal% protein and 61.57 kcal% carbohydrate or on a HFD (Rodent Diet with 60% kcal% fat, D12492; Research Diets, Inc., New Brunswick, NJ, USA) containing 60 kcal% fat, 20.0 kcal% protein and 20 kcal% carbohydrate. Regular monitoring of animals included checks on body weight and appearance (lethargy, breathing, piloerection, hunching, dehydration, gait). Body weight was measured using a Denver Instruments Balance (#SG-601, Fisher Scientific, UK). No unexpected adverse effects that could be linked to procedures were recorded during or after the experiments.

### Mouse genetic modifications

The generation of the RipII-CRE-ER allele (Tg(Ins2-cre)23Herr) and the Ins1-Cre allele (Ins1 ^tm1.1(cre)Thor^) have been previously described ( Herrera, 2000; Thorens *et al.,* 2015). The floxed-STOP SUR1-R1183W allele was generated as follows: cDNA for mouse SUR1 with the R1183W mutation and an extracellular 3-FLAG tag sequence (MDYKDHDGDYKDHDIDYKDDDDK, inserted between V1049 and L1050) was integrated into the genome of C57BL/6N mice using a shuttle system optimized for overexpression studies ( Dolatshad *et al.,* 2015). In brief, SUR1-R1183W-3-FLAG cDNA was targeted to the Rosa26 genomic locus using RS-PhiC embryonic stem (ES) cells, a receiver ES cell line that enables shuttling of desired cDNAs from a matching shuttle vector into the Rosa26 locus via PhiC31 integrase site-directed recombination. The ES cell receiver allele designation is Gt(ROSA)26Sor ^*tm*1( *CAG*- *Hygro*- *PhiC31*) *Wthg*^. The PhiC31 integrase is constitutively expressed by the RS-PhiC ES cells until recombination occurs, at which point the integrase cassette is replaced by the shuttle cassette ( Dolatshad *et al.,* 2015). The main features of the successfully targeted allele are Cre/LoxP-conditional expression of the SUR1-R1183W-FLAG minigene and optional promoter switching: the strong CAG promoter upstream of the minigene can be removed by Flp-recombinase mediated excision, functionally replacing it with the weaker endogenous Rosa26 promoter. RS-PhiC ES cells originate from the C57BL/6N mouse strain and the SUR1-R1183W allele was generated and maintained on a pure C57BL/6N background. RipII-Cre-ER and Ins1-Cre parent animals were congenic backcrosses to the C57BL/6J strain. Thus, all experimental animals were F1 hybrids of B6/N and B6/J.

Successful targeted insertion was detected at the ES cell stage by long-range PCR (upstream primers: AGCCATTGCCTTTTATGGT & GCCCCTCTCAGGTTAATCCCAG, 2843 bp amplicon; downstream primers: TGTGCGCTATGACAGCTCCCTG and CGGGAGAAATGGATATGAAGTACTGGGC, 1557 bp amplicon).; DreamTaq Green PCR Master Mix (2X), #K1081 Thermo Fisher Scientific UK; cycles: 180s 95°C, 40x(60s 95°C, 60s 55°C, 60s 72°C), 300s 72°C; Applied Biosystems 2720 Thermal Cycler, Thermo Fisher Scientific UK). Germ line transmission was tested for by PCR ( Figure 4A) using primers TGACAACTTATCTTCGGTGC & CGCCGTCATGGTCCTTATAG, 206 bp amplicon (same PCR master mix and thermocycler; cycles: 180s 95°C, 40x(60s 95°C, 30s 55°C, 30s 72°C). Additionally, germ line transmission and integration site were independently confirmed using copy number quantitative realtime PCR. This was run as a service by the MRC Mary Lyon Centre, Harwell, UK, using the following probes and primers: wt allele: primers TCCCTCGTGATCTGCAAC, AACGCCCACACACCAGGTTAG, FAM-probe CAGTCTTTCTAGAAGATGGGCGGGA. Neomycin (knock-in allele): primers GGTGGAGAGGCTATTCGGC, GAACACGGCGGCATCAG, FAM-probe TGGGCACAACAGACAATCGGCTG. SUR1 (exon-exon boundary): primers GTGGAGTGGACAGGACTGAAG, GTGCTGGTCAATGGTGTTACAG, FAM-probe CCAAGAGGCTGCACCGCAGC. Dot1 internal control: primers GCCCCAGCACGACCATT, TAGTTGGCATCCTTATGCTTCATC, VIC-probe CCAGCTCTCAAGTCG.

### Electrophysiology

Wild-type or mutant SUR1 were cloned into the pcDNA3.1 vector and transiently co-expressed with pcDNA3.1/Kir6.2 in HEK293 cells, with SUR1 in stoichiometric excess (between 1:5 and 1:30). Currents were recorded from inside-out patches at 20–22°C and -60 mV. The pipette solution contained (mmol/L) 140 KCl, 1.2 MgCl _2_, 2.6 CaCl _2_ and 10 HEPES (pH 7.4 with KOH). The standard internal (bath) solution contained (mmol/L) 107 KCl, 1 CaCl _2_, 2 MgCl _2_, 10 EGTA and 10 HEPES (pH 7.2 with KOH) plus MgATP or gliclazide as indicated. Relative current I/I _0_ as a function of intracellular MgATP concentration [MgATP] _i_ was fit using the equation I/I _0_ = max + (min – max) * [MgATP] _i_^n^ / (k ^n^ + [MgATP] _i_^n^), with max, min, k and n as free-fit parameters using the OriginPro 9.1 software. GnuPlot ( http://www.gnuplot.info/) has been suggested as a free alternative to the Origin software, but the authors have no personal experience concerning its suitability.

### Islet isolation

Mice were killed by cervical dislocation (the recommended schedule one method for this species and developmental stage), the pancreas removed, and islets subjected to liberase digestion. Islets were isolated as described previously ( Brereton *et al.,* 2014; Girard *et al.,* 2009) by handpicking (for insulin secretion and western blotting) or (for western blotting) using a Ficoll-paque gradient (GE Healthcare, Chalfont St Giles, UK), as described ( Cantley *et al.,* 2011).

### Western blotting

For western blot analysis, islets were lysed in detergent buffer (110 mM KOAc, 0.5% v/v Triton X-100, 10 mM HEPES pH 7.4). Extracts were separated on 4–12% SDS–polyacrylamide gels, blotted onto PVDF membranes, and assayed using mouse anti-FLAG antibody (monoclonal M2, Sigma # F3165, 1 ug/mL; 1% w/v skimmed milk powder Sigma #70166 in TBS), HRP-coupled anti-mouse-IgG (NA931V, Amersham, 1:20000 in 2% w/v skimmed milk powder TBS) and high sensitivity ECL detection reagent (Thermo #34095).

### Fed blood glucose, IPGTT & IPITT

Fed blood glucose was monitored regularly from small tail incisions after administration of a local anaesthetic (EMLA cream, Eutectic Mixture of Local Anesthetics, Lidocaine/Prilocaine, AstraZeneca), using Lithium-Heparin microvette tubes (Sarstedt). Plasma glucose was measured using an Analox Glucose Analyser GM9.

IPGTTs were conducted after an overnight fast. Blood glucose was measured by tail incision immediately before, and then 30, 60 and 120 minutes after injection with 2 g of glucose per kg body weight (20% glucose in 0.9% NaCl). An additional time point (15 minutes) was added for the IPGTT at 13 weeks of age.

IPITTs were conducted after a 4h fast. Blood glucose was measured by tail incision immediately before and 15, 30, 45, 60 and 90 minutes after intraperitoneal injection of insulin (0.75 U/kg _BW_).

### Glucose stimulated insulin secretion

Islets were prepared as described ( Brereton *et al.,* 2014; Girard *et al.,* 2009), and cultured overnight in RPMI solution containing 5 mmol/L glucose (Gibco), supplemented with 100 units/mL penicillin and 100 μg/mL streptomycin, at 37°C in a humidified atmosphere of 5% CO2/95% air. After overnight culture, insulin secretion was measured in 1 hour batch incubations (10 islets each) at 37°C in Krebs-Ringer-HEPES buffer (in mmol/L): 120 NaCl, 4.7 KCl, 2.5 CaCl2, 1 KH2PO4, 1.2 MgSO4, 10 HEPES, and 25 NaHCO3, pH 7.4 (with NaOH), plus 0.1% BSA and glucose and glibenclamide as indicated. Before stimulation, islets were pre-incubated with 2 mM glucose for 1 hour in Krebs-Ringer-HEPES buffer. At the end of the experiment, insulin was extracted from islets with acid ethanol (75% v/v EtOH; 15 mM HCL; 0.1% v/v Triton-X100). Insulin was measured using a Mercodia Mouse Insulin ELISA (Mercodia, 10-1247-10).

### Statistics

Error bars show standard error (SEM) and symbols denote the arithmetic mean. Unless otherwise stated, p-values derive from a Welch t-test (null hypothesis: no difference between genotypic group and control littermates). Graphpad PRISM 8, OriginPro 9.1 and Microsoft Excel 2010 were used for statistical analysis and plotting. P values of 0.05 and below were considered significant.

## Data availability

### Underlying data

Figshare: Data supporting phenotype of a transient neonatal diabetes point mutation (SUR1-R1183W) in mice. https://doi.org/10.6084/m9.figshare.10002044

This project contains the following underlying data:

- figure1A version2.xls (non-identifiable patient data used in generation of Figure 1A)
- figure 1B version2.xls (non-identifiable patient data used in generation of Figure 1B)
- figure 2.xls (original electrophysiology data used in Figure 2)
- figure 5A.xls (fed blood glucose data used in Figure 5A)
- figure 5B.xls (intraperitoneal glucose tolerance test data used in Figure 5B)
- figure 6a.xls (body weight data used in Figure 6A)
- figure 6BC.xls (fed blood glucose data used in Figure 6B and 6C)
- figure 7A.xls (intraperitoneal glucose tolerance test data used in Figure 7A)
- figure 7B.xls (intraperitoneal glucose tolerance test data used in Figure 7B)
- figure 7C.xls (intraperitoneal insulin tolerance test data used in Figure 7C)
- figure 7D.xls (original body weight and fasting blood glucose data used in Figure 7D)
- figure 8A.xls (isolated islet insulin secretion data used in Figure 8A)
- figure 8B.xls (isolated islet insulin content data used in Figure 8B)

Data are available under the terms of the Creative Commons Attribution 4.0 International license (CC-BY 4.0).
