# Supplementary material for: Phenotype of a transient neonatal diabetes point mutation (SUR1-R1183W) in mice
Source: Wellcome Open Res. 2021 Mar 15;5:15. Originally published 2020 Jan 30. [Version 2] doi: 10.12688/wellcomeopenres.15529.2 (PMC8323074; doi:10.12688/wellcomeopenres.15529.2)
Supplement: Peer review history

# Peer review: Reviewer response for version 2

**DOI:** 10.21956/wellcomeopenres.17275.r44952

**Published:** 2021-08-18

**Authors:** Heba Hassan

**Affiliations:** 1 Division of Human Genetics and Genome Research, Department of Medical Molecular Genetics, National Research Centre, Cairo, Egypt

In this article, the authors studied (SUR1-R1183W) mutation which is associated with Transient Neonatal Diabetes Mellitus (TNDM). The use of data from the Exeter patient cohort rationalized the aims of the work. Furthermore, studying the mutant HEK293 cells illustrate the severe phenotype. Mouse models were successfully generated using Cre/LoxP technology expressing SUR1-R1183W mutant protein. However, the recapitulation of the human phenotype isn’t elucidated in transgenic mice.

 The work is scientifically sound, the methods and results are clearly presented via illustrating figures and graphs. The references are appropriately cited. The discussion demonstrated a detailed explanation of the potential causes for the pitfalls to recapitulate the human phenotype. The conclusion recommended CRISPER/Cas transgenic mouse model as a less invasive technique. Whereas, Generation of *in vivo* models with homo-mutant and hetero-mutant with 1:1 stoichiometry to demonstrate the phenotype differences outcomes would be a consistent recommendation.

**Minor comment:**

 The heading “Exeter patient cohort data source and analysis” should be moved into the first heading at the methods section to keep the sequence of events.

Is the work clearly and accurately presented and does it cite the current literature?

Yes

If applicable, is the statistical analysis and its interpretation appropriate?

Yes

Are all the source data underlying the results available to ensure full reproducibility?

Yes

Is the study design appropriate and is the work technically sound?

Yes

Are the conclusions drawn adequately supported by the results?

Yes

Are sufficient details of methods and analysis provided to allow replication by others?

Yes

Reviewer Expertise:

Medical Molecular Genetics

I confirm that I have read this submission and believe that I have an appropriate level of expertise to confirm that it is of an acceptable scientific standard.

# Peer review: Reviewer response for version 2

**DOI:** 10.21956/wellcomeopenres.17275.r44953

**Published:** 2021-07-29

**Authors:** István Balogh

**Affiliations:** 1 Division of Clinical Genetics, Department of Laboratory Medicine, Faculty of Medicine, University of Debrecen, Debrecen, Hungary

The paper by Sachse *et al.* describes the generation and analysis of a TNDM point mutation in the ABCC8 gene, in two different model systems. The first system uses HEK293 cell line while the other is a transgenic mice model. 

 Unfortunately, the recapitulation of the human phenotype has been unsuccessful probably due to the mutagenesis system used.

 My major concern is stochiometry of the heterooctamer channel.

 According to the theoretical expression of the wild type/mutant genes, it is very difficult to see how the 1:1 ratio can be achieved - this might be one of the major reasons for the failure of the recapitulation of the human phenotype.

 None of the models being similar to the patients' situation as the HEK293 system is a homozygous genotype and the mice have altogether three copies of the ABCC8 gene, clearly different from the *in vivo*. 

 A major point of the work is that other mutagenesis methods are probably more relevant to use in the future.

 Minor point: the term 'hemizygous' might not accurately describe the genotype of the mice, it should be avoided.

Is the work clearly and accurately presented and does it cite the current literature?

Yes

If applicable, is the statistical analysis and its interpretation appropriate?

Yes

Are all the source data underlying the results available to ensure full reproducibility?

Yes

Is the study design appropriate and is the work technically sound?

Yes

Are the conclusions drawn adequately supported by the results?

Yes

Are sufficient details of methods and analysis provided to allow replication by others?

Yes

Reviewer Expertise:

Clinical laboratory genetics.

I confirm that I have read this submission and believe that I have an appropriate level of expertise to confirm that it is of an acceptable scientific standard.

# Peer review: Reviewer response for version 1

**DOI:** 10.21956/wellcomeopenres.16999.r37752

**Published:** 2020-02-14

**Authors:** Show-Ling Shyng

**Affiliations:** 1 Department of Chemical Physiology and Biochemistry, Oregon Health & Science University, Portland, OR, USA

In this work, Sachse *et al*. generated and characterized transgenic mice that express a mutant SUR1, R1183W, which in humans is associated with Transient Neonatal Diabetes Mellitus (TNDM). The goal was to use the mouse models to understand the mechanisms that underlie remission and relapse of TNDM. The mouse models were created using Cre/LoxP technology to specifically express the mutant in pancreatic β-cells by crossing the het SUR1-R1183W line with Ins1-Cre mice for constitutive expression or RipII-Cre-ER mice for inducible expression. While functional characterization of SUR1-R1183W/Kir6.2 channels heterologously expressed in HEK293 cells confirmed reduced MgATP sensitivity, neither constitutive nor induced expression of the mutant in their mice recapitulated the TNDM phenotype based on results presented. The authors did find that high fat diet resulted in increased body weight and reduced glucose tolerance in female β-R1182W mice. However, based on insulin tolerance tests and insulin secretion assay results, it was concluded that glucose intolerance in these mice are likely due to increased insulin resistance rather than reduced glucose-stimulated insulin secretion. 

 The work is clearly and accurately presented with appropriate literature citation. The experiments as described are technically sound. The authors are commended for the thorough discussion on why the mice did not recapitulate the TNDM phenotype. Overall, the study is a useful documentation for guiding future studies. Below are specific comments

1. The use of β-cell specific mutant expression as a human TNDM model seems to be predicated on the assumption that the disease phenotype results from overactive K _ATP_ channels in β-cells. Since the mutant allele expression is expected in all cells that normally express SUR1, the ideal disease model is clearly a knock-in using endogenous promoter. The authors may want to provide the rationale for why they chose β-cell specific expression.
2. The conclusion that insulin secretion in β-R1183W islets is unaltered was based on 20 mM glucose with or without glibenclamide, which are maximum stimulation. A possibility that these islets may exhibit reduced secretion at lower glucose concentrations should be considered.
3. It was pointed out that all SUR1-R1183W patients are heterozygous. As such, ATP sensitivity may not have been impaired as strongly as seen in homomeric mutant channels. Have the authors tried to measure ATP sensitivity in simulated heterozygous state, i.e. WT:mutant co-expression at 1:1 ratio?
4. Page 7, left column results subheading “Phenotype of iβ-R1183W mice with constitutively active SUR1-R1183W”: This should be revised since iβ-R1183W was used to designate inducible β-cell expression.

Is the work clearly and accurately presented and does it cite the current literature?

Yes

If applicable, is the statistical analysis and its interpretation appropriate?

Yes

Are all the source data underlying the results available to ensure full reproducibility?

Yes

Is the study design appropriate and is the work technically sound?

Yes

Are the conclusions drawn adequately supported by the results?

Yes

Are sufficient details of methods and analysis provided to allow replication by others?

Yes

Reviewer Expertise:

KATP channel structure-function and channelopathies.

I confirm that I have read this submission and believe that I have an appropriate level of expertise to confirm that it is of an acceptable scientific standard.

# Response

**Published:** 2020-02-21

**Authors:** Gregor Sachse

We thank Professor Shyng for her positive review and her valuable comments and suggestions. Our responses are given below:

1) […] The authors may want to provide the rationale for why they chose β-cell specific expression.

*We chose this approach to enable us to determine the beta-cell specific contributions to the diabetic phenotype of the patients.  We have updated the text to indicate this rationale.*

2) […] A possibility that these islets may exhibit reduced secretion at lower glucose concentrations should be considered.

*This is an excellent point. Additional secretion measurements at 5 mM and 7 mM glucose had been planned but could not be realised because of low islet yields. We have updated the discussion to include this information.*

3) […] Have the authors tried to measure ATP sensitivity in simulated heterozygous state, i.e. WT:mutant co-expression at 1:1 ratio?

*No, we did not. Unfortunately, it was challenging to achieve a 1:1 ratio in the HEK cell system that we used for initial assessment and we thought it more appropriate to measure the ATP sensitivity of the K _ATP _channel in  β-cells isolated from β-R1183W and iβ-R1183W mice, which simulate the heterozygous state. However, as we found no difference in glycaemic control or in vivo insulin secretion, this was not performed.*

4) Page 7, left column results subheading “Phenotype of iβ-R1183W mice with constitutively active SUR1-R1183W”: This should be revised since iβ-R1183W was used to designate inducible β-cell expression.

*Thank you for spotting this mistake, which we have corrected in the new version.*
